# Guy's Hospital

**Published:** 1896-12-19

**Authors:** 


### GUY'S HOSPITAL.

### DINNER BY THE TREASURER.

Mr. H. Cosmo Bonsor, M.P., the new treasurer of Guy's Hospital, gave a dinner at the Charing Cross Hotel on December 10th, 1896, to meet the Governors of Guy's Hospital and the staff of the hospital and medical school. This is, we are informed, the first dinner of the kind which has ever taken place in the history of Guy's, and if this indeed be so we hope that it will not be the last. The dinner was given in a charming room, exactly suited by its shape and arrangements for the accommodation of the eighty odd guests invited to be present. All the arrangements for the dinner reflected the greatest credit upon the management. After dinner three toasts were proposed: "The Queen and H.R.H. the President of the Hospital," "Guy's Hospital and Medical School," and "The Chairman." Mr. Cosmo Bonsor has a geniality about him which makes everyone at their ease, and he possesses the further knack of always introducing into his speeches an anecdote, or anecdotes, invariably to the point and usually marked by originality. The Chairman very cleverly got out of making a speech for the second toast by suggesting that he had only to point out the subject matter of the toast, and leave it to Dr. Pye-Smith to make the speech, which he accordingly did, with much force and point. Dr. Wilks, who followed, was as amusing and original as usual, and he very wisely brought out the special value of the gathering by emphasising the fact that that night all causes of differences of every kind had been removed, and the governors and the staff were united as one man in the effort to help Guy's Hospital to take the leading position among the hospitals and medical schools of the world. In addition to the speeches some of the past and present students sang with marked ability, Mr. A. Allport, in a song called "Sister Mary," quite bringing down the house. Mr. P. H. Lulham gave a musical sketch, which was so good in itself, and was delivered with such verve and point, that it might be worth his while to consider whether he could not, by training and practice, develop his undoubted talents to a point which would enable him to fill the void left by poor Corney Grain's death. Mr. A. A. Miller displayed much cleverness in his recitation, which, we understand, was original. Mr. Miller has written a burlesque which is likely to be heard of before long, although it has only been given privately up to the present. We congratulate Guy's upon having such an array of talent among the students, and shall hope to have an opportunity of hearing them again, and doing fuller justice to their undoubted gifts. The dinner was a genuine and deserved success, and was fittingly brought to a close by the following amusing anecdote, related by Dr. Wilks: In the days when the late Mr. E. Cock was a resident, one evening an uproar was created in one of the wards by a patient who had become absolutely uncontrollable. The noise made brought the resident staff, and also some of the honorary staff who happened to be in the hospital, to the ward. All remonstrances failed to pacify the patient, and after the steward and certain porters had been summoned to eject the man from the hospital, the man turned round and pointed to the assembly, it was all very well to be a swell like the physicians or surgeons, or to be paid for their services like the superintendent, the steward, and other officers, but he claimed the right as a patient to play his little game too. "For," said he, turning with triumph to those present, "if it weren't for the patients there wouldn't be no 'orspital at all!"

We are glad to hear that one of the governors present was so impressed with the feeling that something might be done to enable the nurses to have an enjoyable evening that he contemplates inviting them and the executive staff to a dinner in the course of the winter.

